# Revisiting *JMLA* case reports: a publication category for driving innovation in health sciences librarianship

**DOI:** 10.5195/jmla.2025.2099

**Published:** 2025-01-14

**Authors:** Jill T. Boruff, Michelle Kraft, Alexander J. Carroll

**Affiliations:** 1 jill.boruff@mcgill.ca, Co-Lead Editor, *Journal of the Medical Library Association*, Associate Librarian, Schulich Library of Physical Sciences, Life Sciences, and Engineering, McGill University, Montreal, QC, Canada; 2 kraftm@ccf.org, Co-Lead Editor, *Journal of the Medical Library Association*, Medical Library Director, Cleveland Clinic Foundation, Cleveland, OH, United States; 3 alexander.j.carroll@vanderbilt.edu, Associate Editor, *Journal of the Medical Library Association*, Associate Director, Stevenson Science and Engineering Library, Vanderbilt University, Nashville, TN, United States

## Abstract

In the April 2019 issue (Vol. 106 No. 3), the *Journal of the Medical Library Association (JMLA)* debuted its Case Report publication category. In the years following this decision, the Case Reports category has grown into an integral component of *JMLA*. In this editorial, the *JMLA* Editorial Team highlights the value of case reports and outlines strategies authors can use to draft impactful manuscripts for this category.

## INTRODUCTION

In the April 2019 issue (Vol. 106 No. 3), the *Journal of the Medical Library Association (JMLA)* debuted its Case Report publication category [1]. The Case Report category replaced the preceding Case Study category in efforts to delineate the difference between case reports as a publication category and case study as a research method [2]. In the years following this decision, the Case Reports category has grown into an integral component of *JMLA*. Each issue of *JMLA* typically includes between two and four Case Report articles. Topics featured with Case Reports vary, reflecting the breadth of services and initiatives that contemporary health science information professionals engage in across their local communities. Recent issues of *JMLA* have included descriptions of systematic review services, health information literacy programs, internship programs, and virtual conferences, among others.

Prospective *JMLA* authors often have trouble distinguishing whether a manuscript best fits within the Original Investigation or the Case Report category. Table 1 provides an overview of both submission types. While Original Investigations are slightly longer in extent, both submission types feature empirical articles that utilize structured abstracts, structured article formats, and are subject to *JMLA*'s Data Sharing Policy.

**Table 1:** A comparison of Original Investigations and Case Reports

	Original Investigations	Case Reports
**Purpose**	Describe research that employs any type of quantitative or qualitative method of analysis. Examples include intervention studies, surveys, content analyses, qualitative case studies, bibliographic or bibliometric analyses, and search filter development and testing.	Describe the development, implementation, and evaluation of a new service, program, or initiative, typically in a single institution or through a single collaborative effort.
**Structured Abstract Format**	Objective, Methods, Results, Conclusions	Background, Case Presentation, Conclusions
**Structured Article Format**	Introduction, Methods, Results, Discussion	Background, Case Presentation, Discussion
**Extent**	No more than 5,000 words; up to 6 figures and tables	No more than 3,000 words; up to 3 figures and tables
**Data Sharing**	Subject to the *JMLA* Data Sharing Policy	Subject to the *JMLA* Data Sharing Policy

Given the substantive overlap between these two categories, we would like to highlight a few primary points of difference that delineate Original Investigations and Case Reports:

- *The inclusion of research questions:* Original Investigations are research projects launched to answer research questions; they will include research objectives and/or falsifiable hypotheses based on those questions. Case Reports typically describe local initiatives created based on the needs of a specific community; the premises guiding these reports are grounded in professional intuitions and assumptions.
- *A rigorous and well-defined research methodology:* Original Investigations must include a well-documented research methodology. While strong Case Reports include a program evaluation component, these evaluations are often focused on quality improvement rather than hypothesis testing, and may not have a rigorous methodology
- *A goal of generalizability*: Original Investigations attempt to answer broad research questions with results that have generalizable implications for the field. While strong Case Reports will share wider implications for other professionals to consider, Case Reports serve to bring to our attention to novel or surprising initiatives in local contexts that might not otherwise be described in the literature.

Authors sometimes express concerns over having their submissions placed within the Case Report category due to the misconception that Case Reports are a lesser publication type. While Original Investigation articles feature more rigorous research design and more intensive data analysis, Case Reports fill a critical role for *JMLA* readers: they present novel initiatives or provide preliminary findings that drive innovation and advance the practice of health information professionals. Moreover, prior to publication, Case Reports matriculate through the same rigorous double-blind peer review, editorial review, and copyediting processes as Original Investigations. In the remainder of this editorial, we will highlight the impact of recent *JMLA* Case Reports and discuss strategies authors can implement when drafting their own Case Report submissions.

## THE IMPACT OF CASE REPORTS

Case Reports are highly valued by *JMLA*'s readership and widely read by library practitioners and information science researchers. Between September 2023 and September 2024, eight case reports received over 500 full-text views. Of the 100 most viewed *JMLA* articles during this period, 10 of these articles were Case Reports. Coincidentally, the most highly viewed *JMLA* Case Report during this period was Gotschall et al.'s “Journals Accepting Case Reports,” which published a list of over 1,000 journals that publish case reports across dozens of medical specialties [3]. As with health information specialists, medical professionals value case reports as both an information source and a venue for disseminating their work.

The impact of Case Reports extends to citation practices, as well. We pulled citable *JMLA* articles (e.g., Knowledge Syntheses, Original Investigations, Case Reports, and Special Papers) published between 2019–2023 using the Web of Science Core Collection. While the most highly cited papers are knowledge synthesis studies, case reports perform similarly to original investigations in terms of citation impact. The median number of citations for all citable items is 3; the median for case reports is 2. Mean citations for all citable is 6.6 (SD 17.0); mean citations for case reports is 3.0 (SD 3.4). Of the top 40 articles in the last four years, four were published as Case Reports. Far from being an afterthought, well-written *JMLA C*ase Reports on timely topics reach their intended audience and can shape professional practices.

## ELEMENTS OF A STRONG CASE REPORT

Writing over 40 years ago to an audience of cardiologists, DeBakey and DeBakey [4] established several criteria for effective case reports that remain relevant for practitioners today. They contend that case reports should describe “unusual or puzzling features,” depict “new, little known, or rare” occurrences, highlight “unexpected favorable or adverse” outcomes, or identify “possible causal relation, hitherto unreported, between two or more” items. While Case Reports within *JMLA* need not be entirely novel developments, the initiatives described should present a unique set of features, circumstances, or participants that separate them from previously published reports. As argued by DeBakey and DeBakey, valuable case reports “should uncover [a] truly unusual case from which others can learn something new”[4].

As such, Case Reports are not “light” or “easy” versions of Original Investigation articles, which seek to identify generalizable findings. Rather, Case Reports serve the distinctly different purpose of helping health science information professionals learn of surprising or innovative services or initiatives unfolding elsewhere within the field. To this end, Case Reports should describe the institutional setting, stakeholders, and other contextual information in sufficient enough detail for readers to understand the needs of the community from which the new initiative arose and consider whether the initiative could be equally beneficial within their local contexts. An effective Case Report also situates itself by mentioning some of the other possible solutions reported in the literature and making a case for why this novel approach improved upon these previously cataloged alternatives.

While Case Reports might serve as the starting point for encouraging future generalizable original research studies, Case Reports need not feature the same in-depth data collection and analysis that is reported and discussed within Original Investigations. *JMLA* authors are encouraged to describe and report any relevant evaluation data that were gathered for the case. Example evaluation data to present within Case Reports may include attitudinal surveys, usage statistics, or responses from program participants. Inclusion of these data when available can enrich the Case Report, as these data can substantiate authors' claims about implications for professional practice while also establishing baseline findings to be further explored by readers.

However, some Case Reports suffer from paying too much attention to the evaluation process instead of describing the relevant context that made the case novel in the first place. *JMLA* often receives manuscripts that describe new services, programs, or initiatives whose evaluation data includes samples that are too small and non-representative to be meaningful, regardless of the robustness of the data collection and analysis methods used. In these instances, in-depth analysis of insufficiently powered studies may limit the authors' ability to adequately describe and reflect upon the service. While the implications do not have to be generalizable, strong case studies describe the authors' reflections on lessons learned.

In other instances, a Case Report's evaluation strategy may be sparse, but the program underlying the case is novel, important, and described objectively. Authors in these situations may benefit from describing the limitations of their evaluation process instead, rather than attempt to pull insights from such limited pools of data. Sparse data should not keep authors from considering the Case Report as a publication type for their innovative initiatives.

## CONCLUSION

*JMLA* strives to enhance the knowledge base of health science information professionals through the publication of thoughtfully designed journal articles. *JMLA*'s Case Reports contribute to that mission by providing a forum for practicing health sciences librarians to highlight and share exciting programs occurring at their local contexts, regardless of whether these programs are research-based. The *JMLA* editorial team views Case Reports, when effectively written, as a valuable medium for driving innovation within professional practices. We hope this editorial will encourage new and previous *JMLA* authors to reflect on projects currently ongoing at the institutions and consider whether the programs' designs and outcomes may have the makings of a promising Case Report.
